# The Incidence of Antibody-Mediated Rejection Is Age-Related, Plateaus Late After Kidney Transplantation, and Contributes Little to Graft Loss in the Older Recipients

**DOI:** 10.3389/ti.2023.11751

**Published:** 2023-12-22

**Authors:** Michiel G. H. Betjes, Judith Kal-van Gestel, Joke I. Roodnat, Annelies E. de Weerd

**Affiliations:** Rotterdam Transplantation Institute, Department of Nephrology and Transplantation, Erasmus Medical Center, Rotterdam, Netherlands

**Keywords:** kidney transplantation, age, elderly, antibody-mediated rejection, graft survival

## Abstract

It is not known whether antibody-mediated rejection (ABMR) is age-related, whether it plateaus late after transplantation, and to what extent it contributes to graft loss in older recipients. Patients transplanted between 2010 and 2015 (*n* = 1,054) in a single center had regular follow-up until January 2023. Recipients were divided into age groups at transplantation: 18–39 years (“young”), 40–55 years (“middle age”), and >55 years (“elderly”). Ten years after transplantation the cumulative % of recipients with ABMR was 17% in young, 15% in middle age, and 12% in elderly recipients (*p* < 0.001). The cumulative incidence of ABMR increased over time and plateaued 8–10 years after transplantation. In the elderly, with a median follow-up of 7.5 years, on average 30% of the recipients with ABMR died with a functional graft and ABMR contributed only 4% to overall graft loss in this group. These results were cross-validated in a cohort of recipients with >15 years follow-up. Multivariate cox-regression analysis showed that increasing recipient age was independently associated with decreasing risk for ABMR. In conclusion, the cumulative risk for ABMR is age-dependent, plateaus late after transplantation, and contributes little to overall graft loss in older recipients.

## Graphical Abstract

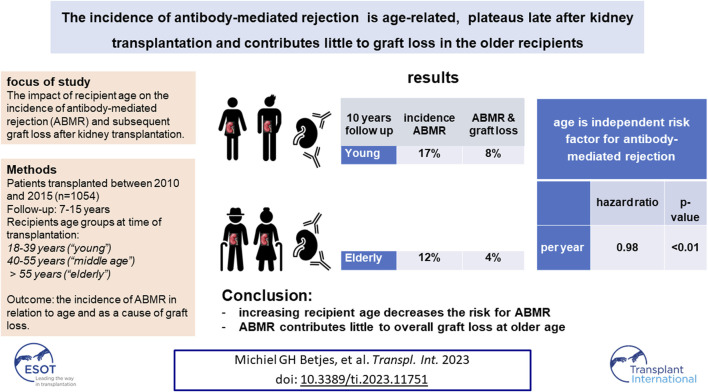

## Introduction

In recent years, the classification of causes of long-term kidney allograft loss other than death have shown a paradigm shift. Chronic allograft nephropathy [1, 2] has been replaced as a concept by redefined and regularly updated pathology criteria (Banff criteria). This includes the categories of (chronic-active) antibody-mediated rejection (ABMR) and interstitial fibrosis with tubular atrophy (IFTA), which are now recognized as major causes of graft loss across all age-categories [3]. With regard to the long-term outcome, in particular, ABMR is now recognized as a major cause of graft loss other than death [4–6].

Unfortunately, published data on histological proven causes of graft loss in the long-term are relatively scarce. Recently, we reported on biopsy results before graft failure occurred in a cohort of recipients with very long-term follow up of at least 15 years up to 24 years [7]. The results showed that death is an important competitive risk factor for (chronic-active) ABM-related graft loss and gave an indication that the cumulative incidence of c-aABMR plateaued out at around 15 years after transplantation. This finding is of interest as it mirrors the plateau in the incidence of TCMR which is usually observed 1–5 years after transplantation, depending on recipient age [8, 9]. This phenomenon is explained by the development of donor-specific hypo responsiveness (DSH) on the level of T cell alloreactivity, which is mediated by activation-induced cell death of donor-specific alloreactive T cells [10, 11]. DSH allows for a lower intensity of immune suppression in the first months after transplantation, as is part of the protocol in the majority of transplantation centers. The occurrence of a parallel DSH for ABMR could be the rationale for a change in immune suppression much longer after transplantation. In addition, increasing age is associated with decreased functionality of the immune system leading to a decreased risk for TCMR [12–15]. Whether age also decreases the risk for ABMR is not known but in our previous study a trend towards a lower cumulative incidence of ABMR in elderly recipients was observed [7]. Such a phenomenon would indicate that in this age group reduction of the immune suppression could be realized at an earlier point in time after transplantation, which will result in less side effects such as infections. As elderly recipients are the fastest growing group of kidney transplant recipients [16], it is important to have knowledge about the risk for ABMR over time after transplantation and the relative contribution to graft loss in this age group.

Given the long-term follow up of our previous study, the initial immune suppressive medication differed from the current standard with tacrolimus, MMF, and steroids, as 35% were treated with ciclosporin instead of tacrolimus and <10% received anti-CD25 induction therapy. Another difference was the relatively low number of elderly recipients (*n* = 230 aged >55 years). For this reason, we analyzed a larger and more recent cohort of recipients transplanted between 2010 and 2015, in order to have at least 7 years of follow-up data. The primary objective was to study the incidence of ABMR over time. We subsequently characterized age of recipient as a variable for ABMR incidence and the relative contribution of ABMR to overall graft loss.

## Patients and Methods

**FIGURE 1:**
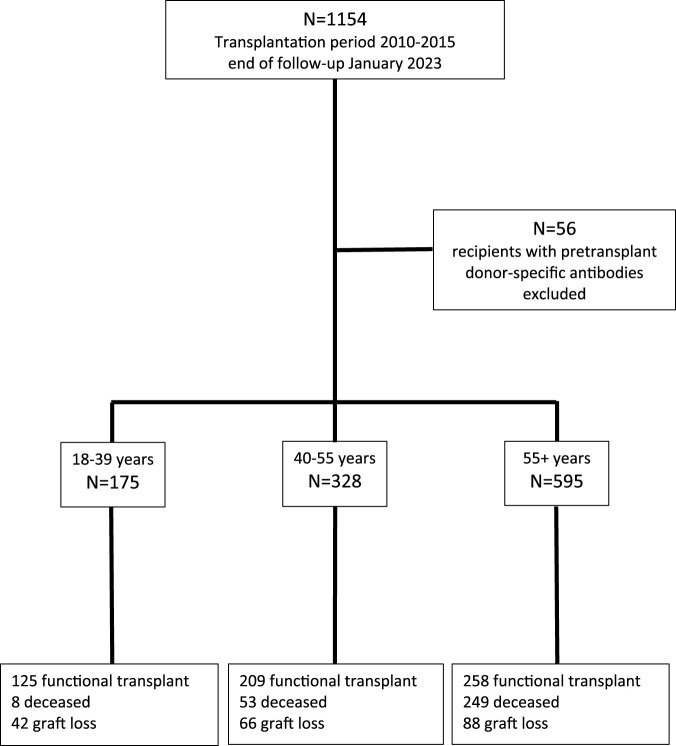
Consecutive recipients of a cross-match negative kidney transplant between 2010 and 2015 and causes of graft loss at the end of follow up at January 2023, according to three different age groups.

This study included all consecutive kidney transplantations performed between January 2010 and December 2015 at the Erasmus Medical Center in the Netherlands. The last follow-up date for data analysis was 1 January 2023. Recipients were seen at least once a year at our out-patient clinic and clinical data were registered in a national database (Netherlands Organ Transplant Registry). Figure 1 shows the flow chart of patients included for analysis. The patients transplanted in the presence of HLA-specific DSA within this period have been described before and were excluded in the current analysis [17].

All other transplantations were performed with a negative complement-dependent cytotoxicity cross-match with both current and historic sera and ABO blood group-incompatibility was not an exclusion criterion. Induction therapy was basiliximab and T cell depletion by anti-thymocyte globulin or alemtuzumab was given in the minority of cases (Table 1). Rituximab was given in cases of ABOi transplantation in combination with IVIG and immune absorption and from 2014 onwards rituximab was replaced by alemtuzumab [18]. The standard immune suppressive medication protocol was based on tacrolimus (aiming for predose concentrations of 10–15 ng/mL in weeks 1–2, 8–12 ng/mL in weeks 3–4, and 5–10 ng/mL, thereafter) combined with mycophenolate mofetil (starting dose of 1 g b.i.d., aiming for predose concentrations of 1.5–3.0 mg/L) and glucocorticoids. All patients received 50 mg prednisolone b.i.d. intravenously on days 0–3. Thereafter, 20 mg oral prednisolone was started and subsequently tapered to 5 mg at month 3 and thereafter stopped within 3 months.

**TABLE 1:** Kidney transplant recipients transplanted in the absence of HLA-specific DSA stratified into three age groups at time of transplantation; period January 2010—December 2015.

	“Young” 18–39 years *n* = 175	“Middle age” 40–55 years *n* = 328	“Elderly” >55 years *n* = 595
Mean age recipient in years (SD)	30.7 (6.0)	48.6 (4.4)	65.3 (5.7)
Mean age donor in years (SD)	47.7 (13.9)	51.2 (13.4)	55.4 (14.6)
Recipient male/female ratio	48/52%	47/53%	45/55%
Follow-up in years, median	9.0	8.8	7.5
Deceased/living donor kidney	22/78%	30/70%	41/59%
DBD type^a^	11%	15%	20%
DCD type^a^	11%	15%	21%
Delayed graft function	11%	17%	23%
Never functioning graft^b^	2%	3%	3%
Pre-emptive transplantation	39%	34%	32%
Cold ischaemia time in hours	4.8 ± 5.7	5.6 ± 6.1	6.8 ± 6.4
Retransplantation	27%	18%	9%
PRA^c^ > 5%	13%	13%	8%
HLA mismatches (median)			
Class I	1	1	1
Class II	1	1	1
Induction therapy	90%	95%	96%
Anti-IL-2 receptor antibody	81%	86%	90%
T cell depleting antibody	5%	5%	2%
Rituximab^d^	5%	5%	4%
Initial maintenance immune suppression			
Steroids	100%	100%	100%
Tacrolimus/ciclosporin	97/1%	96/0%	97/0%
MMF/azathioprine	98/1%	98/1%	99/1%
Everolimus	0%	1%	0%

^a^ DBD, deceased by brain death; DCD, deceased by circulatory death.

^b^ The category “never functioning graft” includes all kidney transplants that have never functioned sufficiently to allow discontinuation of dialysis.

^c^ PRA, panel reactive antibodies (above 5% indicates the presence of cytotoxic anti-HLA antibodies in recipient’s serum).

^d^ Rituximab was given as induction therapy to blood group ABO-incompatible transplantations.

Data of the current cohort were compared with a previous cohort of kidney transplantations at our center in the period 1995–2005 which has been described in detail previously [7, 9]. For comparison with the current study cohort, only kidney transplant recipients without pre-transplant HLA-specific DSA were included (*n* = 573).

The clinical and research activities being reported are consistent with the Principles of the Declaration of Istanbul as outlined in the “Declaration of Istanbul on Organ Trafficking and Transplant Tourism” and in accordance with the declaration of Helsinki. All patients gave written informed consent for participating in the Netherlands Organ Transplant Registry database, and for assessing additional information on DSA measurements, approval by the institutional review board of the Erasmus Medical Center (MEC-2021-0357) was obtained.

All renal biopsies were *for cause* and were performed in cases of progressive loss of graft function. No DSA surveillance or kidney biopsy protocol was in place. The initial biopsy reviews were rescored following the 2018 Banff reference guide [3]. For analysis, biopsies meeting histological criteria for ABMR with or without positive C4d staining, but without detectable DSA, were scored as ABMR for the current study as described in detail previously [19] and used in prior publications [20–22]. All cases of ABMR, as well as the late cases, were treated with pulse methylprednisolone and intravenous immunoglobulins (1–2 g/kg bodyweight) with additional plasmapheresis in early ABMR. Alemtuzumab was administered as second-line treatment in a small number of patients [19].

### Identification of Anti-HLA Donor-Specific Antibodies

In the case of a positive screening, this was followed by antibody identification by SAB assay of either Lifecodes or OneLambda. For the Lifecodes SAB test, data were analyzed using MATCHIT! Antibody software version 1.3.1 (Immucor) and cut-offs were bead-specific in combination with a raw MFI of more than 750. For OneLambda, data were analyzed using HLA FUSION antibody software version 3.4.18 (One Lambda). For the SAB assay, in 56% of recipients a OneLambda kit was used and in 44% an LifeCodes kit.

### Outcomes and Variables

For data analysis, the outcome of the kidney biopsy was further categorized as previously published [7] in five categories: TCMR, ABMR, recurrence kidney disease, diagnosis of *de novo* kidney disease, and interstitial fibrosis with tubulus atrophy (IFTA). In cases of graft failure, the diagnosis of the *for cause* kidney biopsy was used to categorize the type of graft failure if no other clinical event could explain the loss of kidney function.

The other graft loss categories were a clinical diagnosis of cause for graft failure and “unknown” if no biopsy was performed and a clinical diagnosis for allograft failure could not be made (Table 2). Primary non-function is the category of grafts that never had function after transplantation due to (histological or clinical suspected) acute tubular necrosis (ATN).

**TABLE 2:** Kidney allograft outcomes, according to recipient age at time of transplantation, at follow-up January 2023.

	“Young” 18–39 years *n* = 175	“Middle age” 40–55 years *n* = 328	“Elderly” >55 years *n* = 595	*p*-value
Death with functioning graft	8 (4.6%)	51 (15.5%)	250 (42.0%)	<0.001
Number of graft loss other than death	42 (24.0%)	66 (20.0%)	88 (14.8%)	<0.001
Cause of graft loss				
All rejections	27 (64.3%)	37 (56.1%)	34 (38.6%)	<0.001
TCMR^a^	13 (30.9%)	11 (16.7%)	10 (11.3%)	<0.01
ABMR^a^	14 (33.3%)	26 (39.4%)	24 (27.2%)	ns
Interstitial fibrosis/tubulus atrophy	4 (9.4%)	3 (4.5%)	8 (9.1%)	ns
Recurrence of original disease	4 (9.5%)	7 (10.6%)	10 (11.4%)	ns
Kidney injury/disease^b^	3 (7.2%)	5 (7.6%)	18 (20.4%)	ns
Peri-operative complications	3 (7.1%)	(9.1%)	5 (5.7%)	ns
Unknown	1 (2.4%)	7 (10.6%)	9 (10.2%)	ns
Primary non-function	0 (0.0%)	1 (1.5%)	4 (4.5%)	ns

^a^ TCMR, T cell-mediated rejection; ABMR, antibody-mediated rejection.

^b^ Kidney injury/disease is the category including events or diseases causing irreversible kidney injury leading to graft loss, the category “unknown” indicates that no kidney biopsy was performed and no clinical cause of graft loss was established. ns, not significant (*p* > 0.05).

Three age groups were established based on recipient age at time of transplantation:18–39 years (“young age”), 40–55 years (“middle age”), and >55 years old (“elderly”).

### Statistical Analysis

Differences in patient, donor, and transplant characteristics were assessed by the Fisher’s exact test for categorical variables and Mann-Whitney U test for continuous variables. All *p*-values were 2-tailed.

Death censored graft loss and incidence of graft loss according to cause were assessed by Kaplan-Meier survival analysis with log-rank statistics for differences between strata. Univariate Cox proportional hazards analysis was used to identify clinical and demographic variables as given in Table 1 for association with rejection and graft survival. Variables with a *p*-value of <0.1 were considered for stepwise forward regression to calculate hazard ratios and corresponding confidence intervals. Interaction terms that met statistical significance (*p* < 0.05) were included in the multivariate model. Statistical analysis was performed with software IBM SPSS statistics 21.

## Results

### Baseline Characteristics and Graft and Recipient Survival Per Age Category

The majority of recipients are within the elderly group, with a median age of 65 years, which is in line with the general trend of increasing numbers of elderly patients receiving a kidney transplant (Table 1). Over 90% of recipients were treated with the current standard protocol of immune suppression consisting of anti-CD25 induction followed by triple immune suppression with tacrolimus as calcineurin inhibitor of choice. Known age-related differences in type of donor kidney (fewer living donor kidneys in the elderly), number of re-transplantations (more in the young patients) are also present in this study cohort (Table 1). With the growing number of elderly patients being transplanted, follow-up differed per age group and the medians were, respectively, 9.0, 8.8, and 7.5 years after transplantation for young, middle age, and elderly recipients. At last follow up in January 2023, 71.4%, 63.7%, and 43.3% of young, middle age, and elderly recipients, respectively, were alive with a functioning graft (Figure 1). The frequency of death with a functioning graft ranged from 4.6% in the young to 41.8% in the elderly (Table 2). For graft loss other than death (24.0% for young, 20% for middle age, and 14.6% for elderly recipients), rejection constituted the major cause of graft loss in every age group; 64.3% in the young, 56.1% in the middle age, and 38.6% in the elderly group (Table 2). The frequency of graft loss categorized as “unknown” was low in all age groups and was on average 1.2%.

As expected [23], malignancies, infection, and cardiovascular disease constituted the three main causes of death at follow-up in all age groups with a dominance of cardiovascular disease in elderly patients (Supplementary Table S1).

### The incidence of ABMR plateaus at 10 years after transplantation and is negatively associated with age of the recipient

The total number of recipients with a diagnosis of ABMR was 135 and the cumulative risk for AMBR increased steadily until about 8–10 years after transplantation, after which only very few new cases were diagnosed. HLA-specific DSA were detected in the serum at the time of diagnosis in 53% of cases with ABMR histology (71% in the young, 49% in the middle age, and 58% in the elderly group, *p* > 0.1 for difference between groups). The % of C4d positivity in the biopsies diagnosed as ABMR was on average 43% with also no differences related to age.

**FIGURE 2:**
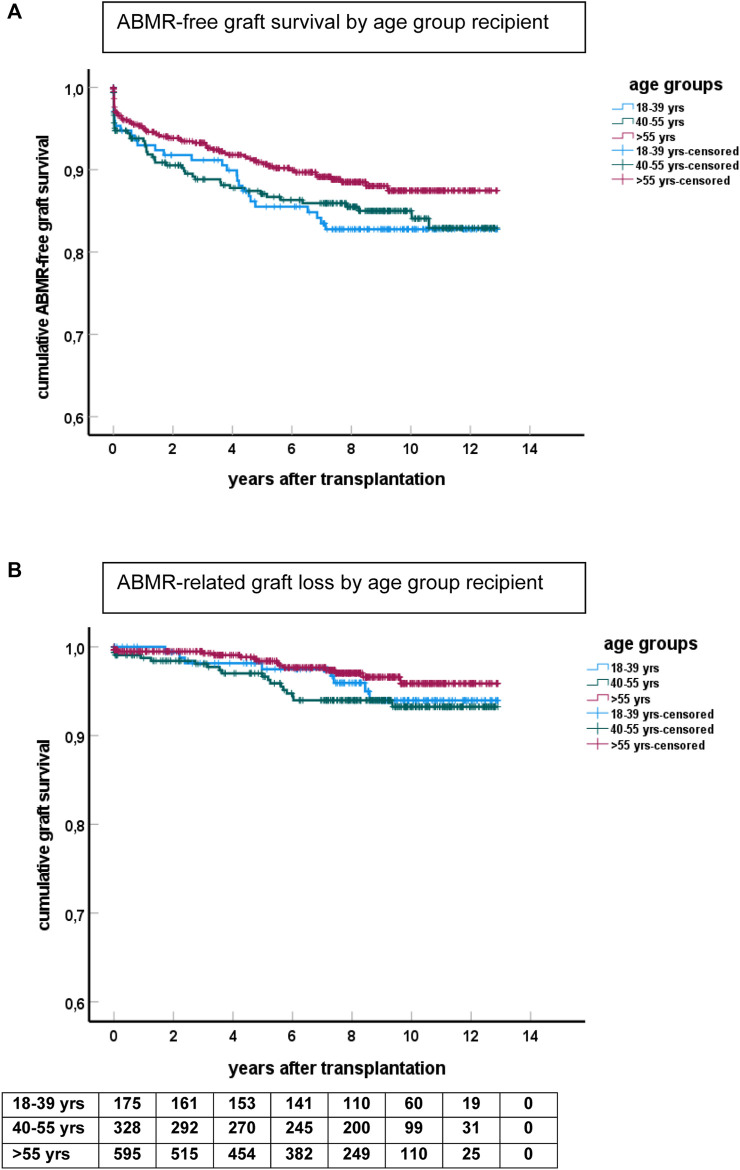
Kaplan-Meier analysis of ABMR and graft survival for different age groups. **(A)** Shows ABMR-free graft survival (*p* = 0.06 for difference between groups) and **(B)** the survival curves for ABMR-related graft loss censored for death and other causes of graft failure. The numbers of recipients in follow-up at different time points after transplantation are shown beneath the bottom figure.

The cumulative incidence of ABMR at 10 years after transplantation was 12% in the elderly, 15% in the middle age, and 17% in the young group (Figure 2). The cumulative incidence per 10 years of recipient age showed only a marginal difference for age groups 55–64 and 65 years or older (Supplementary Figure S1). The average yearly incidence of AMBR between 1 and 6 years after transplantation was 1.1%. The relationship between recipient age and ABMR incidence was confirmed by uni- and multivariate logistic regression analysis (Table 3). Apart from the recipient’s age, the number of HLA-DR mismatches, PRA positivity, and type of donor remained significantly associated with the incidence of ABMR in multivariate Cox regression analysis.

**TABLE 3:** Antibody-mediated rejection after kidney transplantation: uni- and multivariate Cox regression analysis.

	Univariate analysis		Multivariate analysis	
	HR (95% CI)	*p*-value	HR (95% CI)	*p*-value
Age recipient per year	0.98 (0.97–0.99)	0.025	0.98 (0.97–0.99)	0.009
Age donor per year	1.01 (0.99–1.01)	0.35	—	—
Kidney donor DD vs. LD^a^	1.48 (1.05–2.09)	0.024	1.61 (1.12–2.30)	0.009
Cold ischemia time in hours	1.03 (1.01–1.06)	0.007	—	—
T cell depletion induction	1.00 (1.00–1.00)	0.257	—	—
PRA positive^b^	3.08 (2.06–4.60)	<0.001	2.69 (1.76–4.12)	<0.001
HLA class I mismatches	1.20 (0.94–1.53)	0.13	—	—
HLA class II mismatches	1.29 (1.01–1.65)	0.039	—	—
Total HLA class I and II mismatches	1.11 (1.01–1.24)	0.038	1.19 (1.06–1.34)	0.002

^a^ DD, deceased donor; LD, living donor.

^b^ PRA, panel-reactive antibodies in CDC-screening above 5%.

**FIGURE 3:**
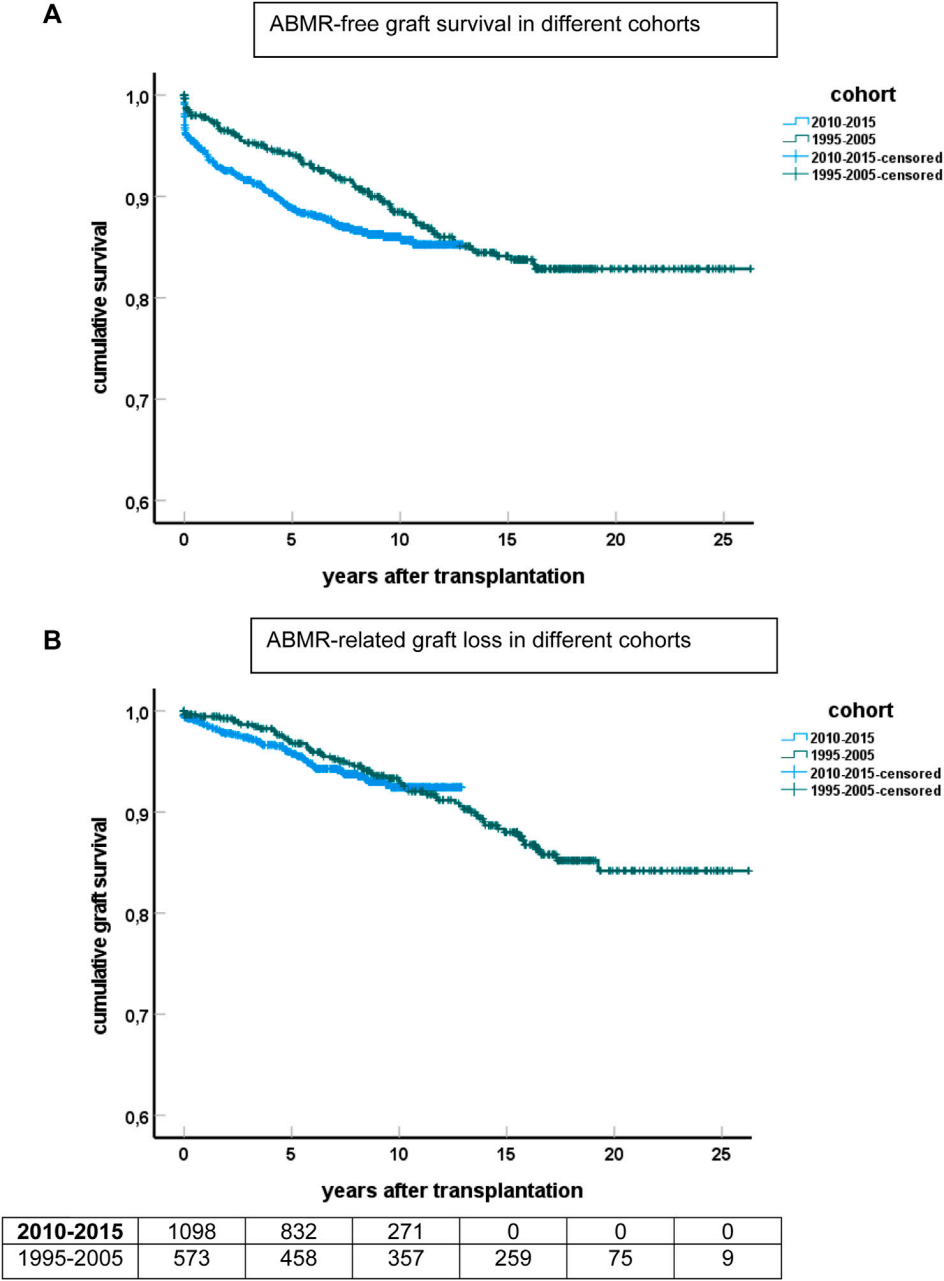
Kaplan-Meier analysis of ABMR and graft survival for different cohorts of recipients. **(A)** Shows ABMR-free graft survival and **(B)** the survival curves for ABMR-related graft loss censored for death and other causes of graft failure. The numbers of recipients in follow-up at different time points after transplantation are shown beneath the bottom figure.

ABMR-related graft loss was recorded in 64 cases (24 elderly recipients, of which one was related to nivolumab and lowering immune suppression because of cancer). At last follow-up, 8%, 7.9%, and 4% of young, middle age, and elderly patients, respectively, had lost their graft because of ABMR (*p* = 0.04 young vs. elderly recipients). Age had no effect on the cumulative graft loss in DSA or C4d positive subgroups of ABMR (as analyzed by KM curves of the age groups and logistic regression analysis, data not shown) Subsequently, ABMR incidence and ABMR-related graft loss were compared with a previously published cohort with a minimum follow-up of 15 years [7]. The study cohort transplanted between 2010–2015 showed a relatively earlier time of diagnosis of ABMR. Incidence of ABMR plateaued at 10 years after transplantation in the more recent 2010–2015 cohort versus 15 years in the 1995–2005 cohort, with cumulative incidence of ABMR of respectively 15% and 12% at 10 years post-transplantation (Figure 3). ABMR-free graft survival showed a remarkable comparable pattern in the elderly in the two different study cohorts (Supplementary Figure S1).

The Kaplan-Meier curves for ABMR-related graft loss for the cohorts 1995–2005 and 2010–2015 practically overlapped (Figure 3).

### Graft Survival of ABMR Cases Is Poor, But Infrequently the Cause of Graft Loss in the Elderly

**FIGURE 4:**
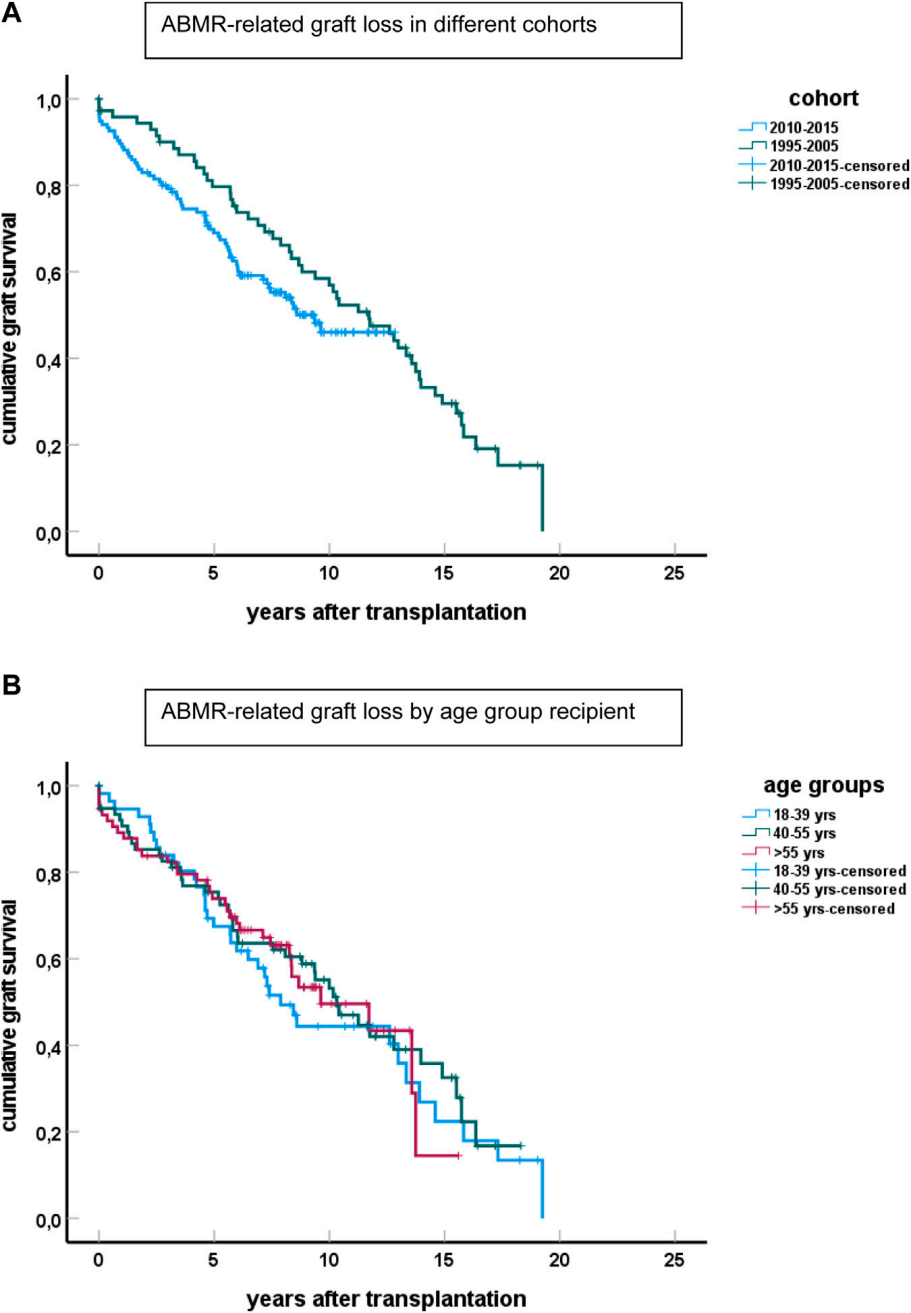
Graft survival of cases diagnosed with ABMR censored for other causes of graft loss than ABMR for the different cohorts is shown in **(A)** (*p* > 0.1 for difference between groups). The number of cases with ABMR in the cohort 1995–2005 is 98 and in the 2010–2015 cohort 135. **(B)** Shows the graft loss because of ABMR for the cases in the different age groups, combining cases from both cohorts.

Graft survival of cases with ABMR was similarly poor for both cohorts (Figure 4) and indicated that >80 percent of cases with ABMR will eventually lose their graft because of ABMR. No significant differences were observed among the different age groups (Figure 4B).

**FIGURE 5:**
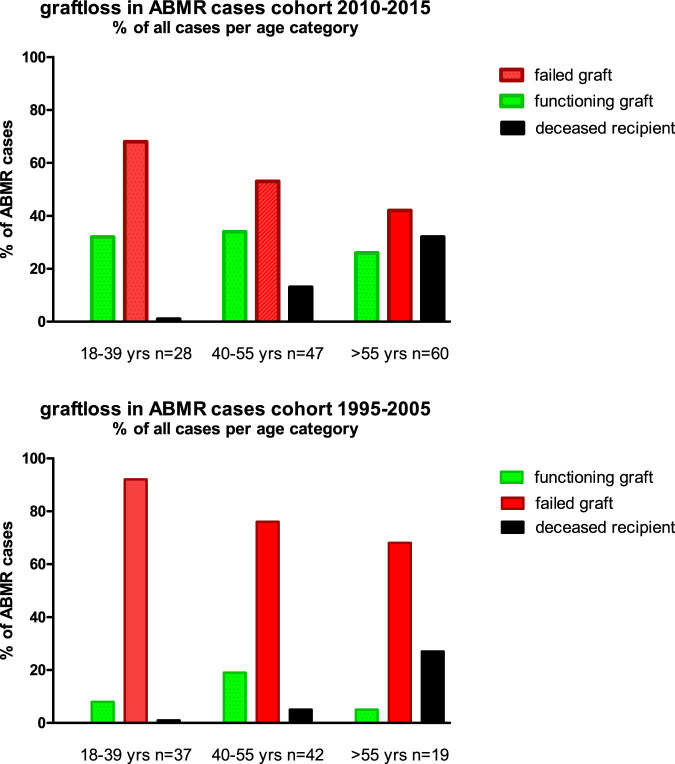
The cause of graft failure of cases with ABMR within the different age groups and cohorts is given. The follow up for the 1995–2005 cohort is at least 15 years (maximum 24 years) and for the 2010–2015 cohort the minimum follow up is 7 years (maximum 13 years).

In the elderly age category, death with functioning graft is a major competing risk, and about 30% of all cases with ABMR died with a functioning graft (Figure 5). This is in sharp contrast to the young recipients with ABMR who will eventually all lose their graft because of ABMR, as is evident from the 1995–2005 cohort with a follow up of at least 15 years.

## Discussion

The major findings in this study are the relationship between the decreased risk for ABMR with increasing age of the recipient, the plateauing of the cumulative incidence of ABMR, and the overall small contribution of ABMR to overall graft loss in the older recipients.

The finding of the age-dependent risk of ABMR requires a large cohort of recipients followed over a long period of time after transplantation, given the relatively low yearly incidence of ABMR. Furthermore, regular follow-up of recipients and a rigorous kidney biopsy protocol should be in place to establish the reason for progressive kidney function decline. The finding of a decreased cumulative incidence of ABMR with climbing age is not unexpected, as immunological aging is related to a less vigorous immune response [24, 25]. ABMR is thought to be primarily caused by *de novo* antibody formation against donor antigens, and immunological aging leads to a decrease in antibody formation which may be due to both impaired T and B cell responsiveness [26, 27].

Given the constant exposure of allo-antigens to the immune system of the recipient, the risk of development of donor-specific antibodies seems to be continuously present. However, DSA formation shows plateauing of the cumulative incidence of serum DSA [28, 29]. This phenomenon parallels the observation in the current study that ABMR incidence also shows evidence of plateauing [28, 29]. Of interest is the observation that the curves of ABMR-free survival have shifted more to an early time of diagnosis in the recent cohort as compared to the earlier cohort from 1995 to 2005. In contrast, the curves for ABMR-related graft loss appear to be very similar between both cohorts. An explanation for these findings could be that in the last decade a kidney biopsy is being considered at an earlier stage of graft deterioration as the awareness of ABMR has increased. However, given the overlapping graft survival of cases with ABMR, it seems likely that the underlying dynamics of ABMR development for both cohorts is comparable. In addition, the graft survival of ABMR cases indicated that given time, most grafts with ABMR will fail because of ABMR after censoring for death.

In the elderly, ABMR contributed to all graft loss for only 4%. This low percentage is largely explained by death as a competitive risk factor, not only because elderly recipients die before they can develop ABMR, but also because in 30% of elderly patients with ABMR, death occurs before ABMR could have led to graft failure.

Similar to other age categories, the cumulative incidence of ABMR in the elderly plateaus at about 8–10 years after transplantation. Similarly to TCMR, where the plateau is reached much earlier after transplantation [8], only a certain proportion of recipients will develop ABMR. Evidently, the presence of mismatches on HL-DR/DQ is an important risk factor for DSA development [30]. The mechanism by which most recipients do not develop ABMR, even after many years of exposing allogeneic HLA molecules to their immune system, has not been elucidated. Given the low contribution of ABMR-related graft loss to overall graft loss in the elderly, in combination with the current lack of precision tools to predict ABMR, it seems that modern immune suppressive drug regimens are sufficient in the majority of elderly patients to protect them from ABMR-related graft loss. However, this observation also raises important clinical questions such as to what extent the intensity of the immune suppressive drugs could be lowered, specifically in the long term. Such a question is important in elderly recipients with an aged immune system, who are prone to infectious, metabolic, and cardiovascular side effects of immune suppressive medication [31–33].

In a recent study, immunological low-risk patients, of whom the majority were elderly, were randomized for continuing standard immune suppression vs. tacrolimus monotherapy at 1 year after transplantation [34]. This feasibility study showed that after discontinuation of MMF, DSA remained undetectable for at least 4 years, infections were reduced, and vaccination responses to SARS-CoV-19 were superior [35]. These data are at least encouraging as it appears feasible to lower immune suppression in selected groups of recipients without increasing the risk for ABMR. However, long term follow-up (until, at least, plateauing of ABMR has been reached) are not yet available.

A potential limitation of the study is the generalizability, as treatment protocols may differ between centers and, for example, prednisone withdrawal at 3 months is not standard in all centers. In addition, the incidence of (subclinical) ABMR would likely have been higher when protocol biopsies would have been taken (for instance based on a DSA surveillance protocol). To what extent this would have changed our conclusions is unclear, especially as there is no proven effective treatment for late ABMR. Another major potential confounder is medication adherence, which may be worse in the younger recipients leading to a higher incidence of ABMR. Unfortunately, the database did not include regular measurements of immune suppressive drugs through levels or a validated medication adherence questionnaire (e.g., BAASIS) to account for this confounder. In a previous study, using the BAASIS questionnaire, no significant effect of recipient age was found on self-reported non-adherence [36].

In conclusion, the incidence of ABMR plateaus at around 10 years after kidney transplantation in an era using the modern immune suppressive medication regime. Increasing recipient age is independently associated with a lower risk for ABMR, and death is a major competitive risk factor for ABMR-related graft loss in the elderly. For these reasons, ABMR contributes relatively little to overall graft loss in the long-term in elderly recipients.

## Data Availability

The original contributions presented in the study are included in the article/Supplementary Material, further inquiries can be directed to the corresponding author.

## Supplementary Material

Supplementary Figure 1
**(A)** The antibody-mediated rejection (ABMR) free graft survival in different cohorts of age at time of transplantation. The green line depicts the data from the cohort 1995-2005 and the blue line from the cohort 2010-2015. Graft loss is a censored event and shown by small crosses. **(B)** The cumulative incidence of antibody-mediated rejection (ABMR) over a period of 10 years in different cohorts of age at time of transplantation. Data derived from the 2010-2015 cohort are shown.

## Abbreviations

ABMR, antibody-mediated rejection; CDC, complement dependent cytotoxicity; C4d, complement C4d; DSA, donor-specific antibodies; HLA, human leukocyte antigen; IFTA, interstitial fibrosis and tubular atrophy; PRA, panel reactive antibodies; TCMR, T cell-mediated rejection.
