# Supplementary figures and images for: The Incidence of Antibody-Mediated Rejection Is Age-Related, Plateaus Late After Kidney Transplantation, and Contributes Little to Graft Loss in the Older Recipients
Source: Transpl Int. 2023 Dec 22;36:11751. doi: 10.3389/ti.2023.11751 (PMC10768842; doi:10.3389/ti.2023.11751)

Supplemental Figure 1A

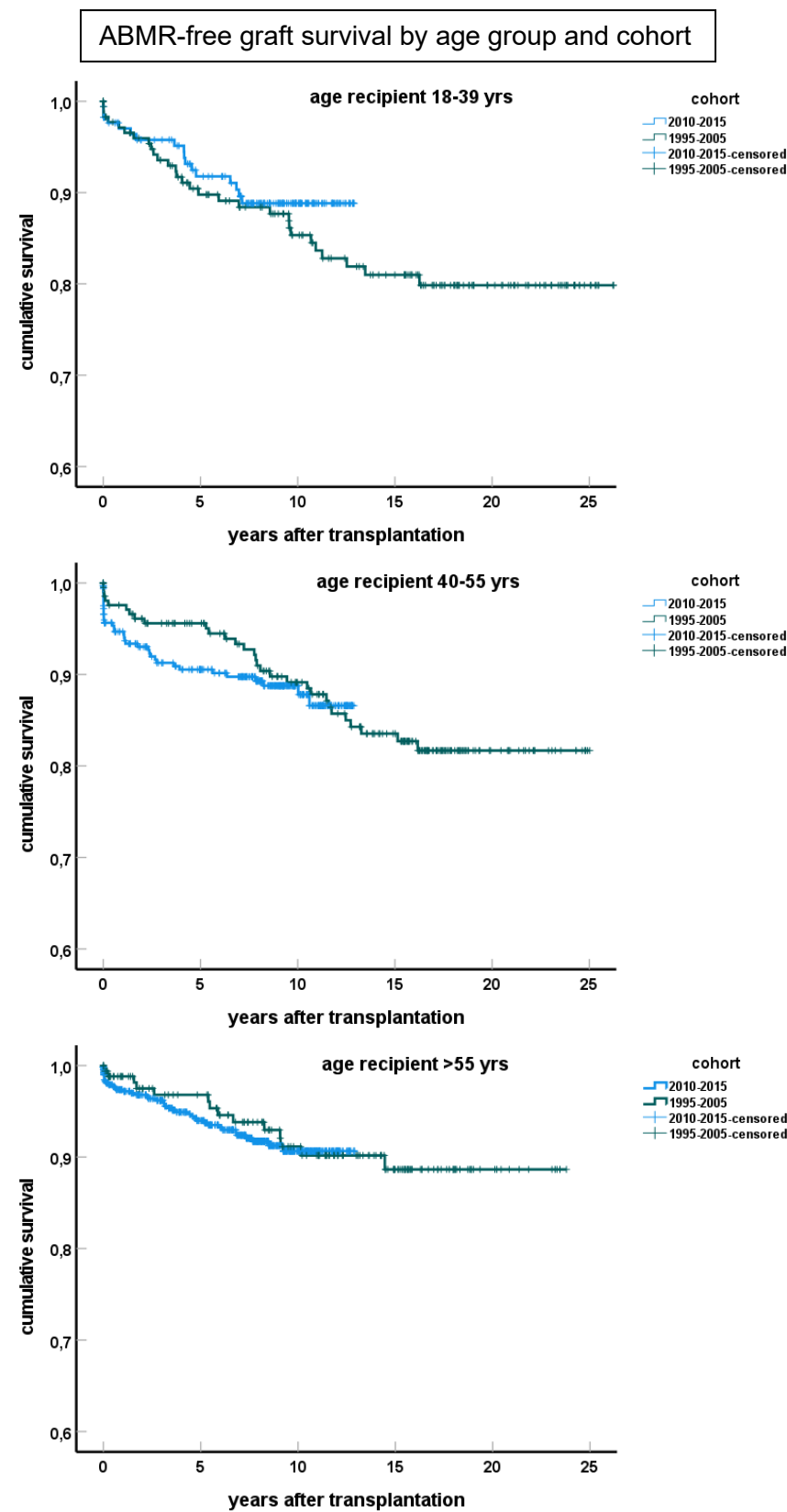

Supplemental Figure 1B

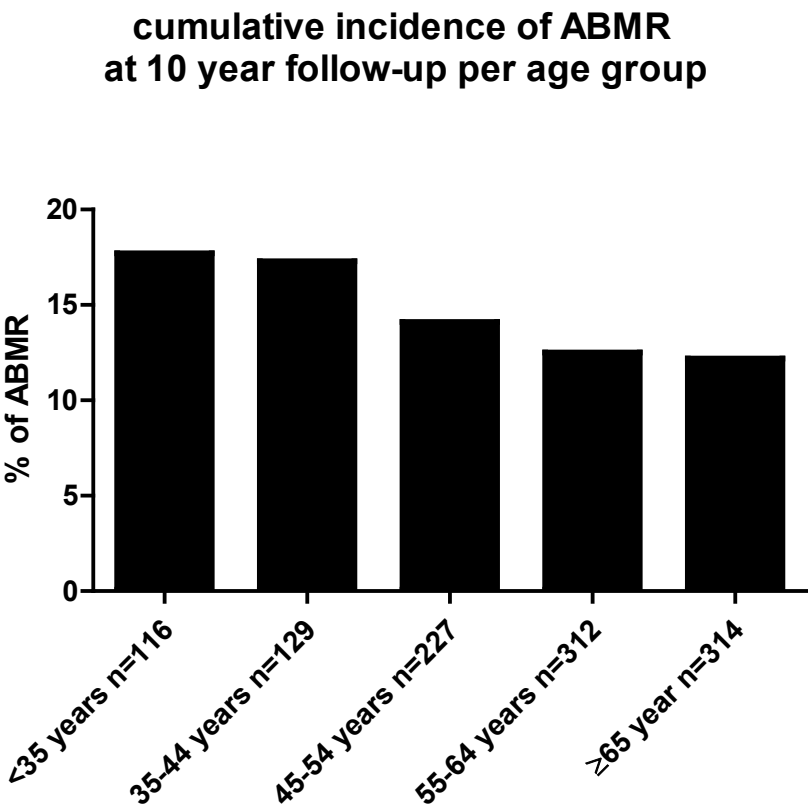

Supplement: Supplementary file 1 [file Image1.pdf]
